# Rachel Challice's Vow

**Published:** 1888-01-28

**Authors:** Lina Nevill

**Affiliations:** Author of "A Future on Trust," "Juno," &c.


### RACHEL CHALLICE'S VOW.

By LINA NEVILL, Author of "A Future on Trust," "Juno," &c.

CHAPTER V.—A WHITE LIE.

"Thus I perceived my sinking spirits fail,
Thus, trembling, I surveyed the gloomy vale,
As near the moment of decision drew."—*Dante.*

"I AM going away, David."

The words fell abruptly from Rachel's lips, and seemed to convey no definite meaning to her auditor. He only glanced at her with momentary surprise.

"Don't go away yet, Rachel. We have only just come up the hill, and you said you wanted to sit down and have a talk. What makes you change your mind so sudden? There wasn't much good in climbing up all this way, just to go there and back again."

"I did not mean that, David; I do want to talk to you still. But I'm thinking of leaving Aberelwyn."

David still seemed to remain stupidly dense.

Going away, in the estimation of the sober, stay-at-home people of Aberelwyn, was an event of rare occurrence; a sort of Hegira, from which the individuals concerned dated previous and subsequent events; a grave undertaking not to be entered lightly upon, but only after mature consideration and preparation.

"Leaving Aberelwyn," he repeated, with vague, parrot-like imitation of her statement.

Then suddenly he faced round sharply; a new light dawning upon him.

"You're not going to get married, Rachel?"

The inquiry seemed almost mockery to the poor girl in her present excited, highly-wrought state of mind.

Marriage for her! No; she left that for others. Nothing for her but voluntary exile—a gloomy, loveless future, shut out from the one joy that might make life at last worth the having.

"Are you going to get married, Rachel?" reiterated David, impatient at her silence.

She roused herself. It was time now to begin to play her self-allotted part.

"Married! Me going to get married! Nonsense, David; what put such an idea into your head?"

"Only you said you were going away."

"And can't a body go away for a bit without being married?"

"Not a young woman like you, Rachel, with no call to do anything but stop quiet at home, as you've been used to do all your life."

"And that is just the very reason why I want to go," she replied shortly. "I've been cooped up at Aberelwyn all my life. I want to get away a bit now, and see what the rest of the world is like."

"It's a bad place for young women to go about alone in," he replied sententiously. "Unless you mean to go to service, and I don't think you'd much like that, after you've been free all these years to come and go as you like. Best stop at home, Rachel, my girl, and go on as you've been used to do."

"It won't hurt *me*," she said scornfully, yet with a tinge of bitterness in her voice. "The world won't hurt *me*. No one will notice me; I am old and ugly. Now, if it was Esther it would be different. She is so pretty, it might be dangerous for her."

She made her comparison with a vague, eager sort of longing to see whether he would contradict or agree with her—a kind of test by which to decide which of the two he considered most attractive.

It was mere fishing for compliments; and poor Rachel had no better luck than most others who go in for that precarious and unprofitable form of nourishment to their vanity.

"Yes," replied David in the most matter-of-fact way. "You are right: it would be worse for Esther. You'd fare better in the world than she, Rachel."

The momentary gleam of hope died down in the elder girl's heart. So, after all, as she used to think, David held her cheap; her idea of his new-born liking for her was a delusion of her vanity, a Will-o'-the-Wisp of her own creation. Esther was, as formerly, far more perfect and precious in his eyes; his love had never wavered from his first ideal, as lately she had begun to suspect.

Her foolishly sensitive mind writhed under the idea of how nearly she had betrayed her unsought love for this man; and the feeling goaded her on to prove to him in return how little she thought about him.

"I shall be glad to get away," she remarked carelessly. "I am tired of everyone here; and Esther will be marrying soon, and won't want me any longer."

She glanced at him to see if he looked conscious under this insinuation, but his always slightly expressionless face remained immovable.

"Well, Rachael," was all he said, "if you are so tired of your friends, the sooner you go the better."

"I think so too," she retorted. "I'll just wait to see Esther married first, and then I'll be off."

"And how soon will that be?"

"You ought to know."

He laughed.

"I'm very sorry, Rachel, but Esther's future husband has not taken me into his confidence. All this is news to me. I don't even know who is the happy man."

Rachel clenched her hands and bit her lip with vexation. How stupid she was! How utterly she was mismanaging the whole matter! If she could do no better than this, she thought, she would spoil all Esther's chances in another manner, and frighten away David Stephens with the ghost of an impromptu, make-believe lover.

"I think you misunderstood me," she said quickly. "I didn't mean that Esther's marriage was a settled thing. What I meant was that she was so pretty and winning, someone is sure to want her before long."

"Very likely," returned the supposed ardent adorer indifferently.

"I sometimes think you admire Esther," she began cautiously.

"She's a pretty little thing certainly. Yes; I'll not deny I consider her that."

"But you do like her, don't you, David?"

"Oh, yes; she's fun enough sometimes, but she's too flighty for me at others."

Esther's recent treatment of him, when she threw him over all one evening, while she flirted desperately with young Morgan Lloyd, of Glaspant Farm, a young man whom no respectable girl should look at, rankled unpleasantly in his mind, and made him chary in his commendation.

"She is very young, you know," pleaded Rachel.

"Well, that is a fault that will mend by time—if being young *is* a fault. Most people count it a blessing; especially those who haven't got it," he concluded drily.

"She'll sober down in time," went on Esther's trumpeter, with desperate energy.

"It's to be hoped she will."

This was discouraging. David was either purposely affecting an indifference he did not feel, or else he was by no means so much enamoured with pretty little Esther Challice as Esther's partial sister supposed.

"It will be a happy man who wins Esther."

"That's as people count happiness. A pretty face is not everything. A wife to my mind would be one who would stay at home, and count her husband above everything."

"Esther would do that," broke out Rachel impulsively.

"Perhaps; but I haven't asked her yet," replied David imperturbably.

He looked straight into Rachel's face as he spoke, and watched keenly every change in her expression, as she sat on the fallen boulder, her hands clasped in her lap, her figure bent slightly forward, and her eyes big and bright with eagerness. A red glow lit up the earth, as though the dying sun were staining the land with his ebbing life-blood, bathing sea, sky, and earth in the same crimson flood. Arran, Snowdon, and Hebog were glowing warm in the lurid light, and the streak of sea out towards the Menai Straits and Anglesea shone a line of vivid molten fire on the far horizon.

Down in Bethgelert and the valley of the Glasslyn the red glow was fading, and shadows of evening crept swiftly up, hiding in a purple veil what a minute before had been so warmly lustrous.

The dashing of the Colwyn river, the tinkle of the sheep-bells, and the cry of some bird alone broke the evening stillness, as Rachel and David sat facing each other on the lonely hill-top.

"Why are you going away, Rachel?"

"For change," she replied lightly, though she longed to tell him the truth; then, once told, let him decide which of the two it should be—the elder or the younger.

But her vow stood a stern, impenetrable fact, barring the way before her.

"I am sorry you are going, Rachel. I shall miss you."

"But you will have Esther."

"Esther!" he exclaimed petulantly. "Why are you always thrusting Esther upon me? Do you never think of yourself?"

She bent her head lower, that he should not see the red flush of joy that flooded her face; and a wild spasm of joy shot through her.

So he did think about her—cared something for her after all——

"It is about yourself I want to talk this evening, Rachel. I must know what you mean about going away. I don't like the idea; I want you here."

"People can't have all they want in this world," she replied, taking refuge in truisms.

"But it's often their own fault when they don't. I'm not one of those to let slip a thing only for the want of laying hold on it. Rachel, tell me the truth—why are you going away?"

"Don't ask me, David," she said faintly, her courage wavering as trial and temptation grew stronger. I can never tell you; but I *must* go."

"There is something about this I can't make out," he returned doggedly. "You have some reason for going which you won't say. It can't be anything to do with Esther, for you don't talk of taking her. You don't mean to say you are going to leave that giddy girl here alone. She's not fit to be trusted, Rachel. You must stay at home, if only to look after her."

He was exerting all his powers of persuasion. Man-like, the moment there was the chance of losing a thing he was wild to retain it. Had Rachel Challice stayed on quietly all her life at Aberelwyn, David Stephens would possibly never have made up his mind to marry her. In all probability, had he chosen either of the sisters, in the teeth of his father's prohibition, he would, from sheer contrariety—because Esther was the most strictly interdicted—have married her.

But now that Rachel was going away, perhaps for ever, she suddenly became a necessity in his life. All visions of Esther vanished, and became tame and distasteful; while to win Rachel Challice's love seemed the one thing needful, worth loss of fortune, the ban of his father's displeasure, the consciousness of making what people would call a bad match. In fact, the possession of Rachel would compensate for every other drawback.

"I shall not leave Esther alone, David," said Rachel. "I will not go till she is in someone's charge who will take care of her. David, why don't you marry her? I am sure you have liked her for months past—long before Granny died; and she loves you dearly."

It was a bold stroke—a kill or cure. Rachel with a steady hand threw down her trump card, and waited breathlessly for the result.

"Because," replied David slowly, "I don't care so much for Esther now. I found I was mistaken in her. It is you, not her, I want to marry, Rachel."

The sudden and unexpected revulsion from hopeless despair to ungovernable joy almost overpowered the girl.

It was the first time in her whole life that she had ever found herself valued and appreciated.

"You want to marry me, David?" she said, almost incredulously. "What put such an idea into your head?"

"It has been there for some time now. I have watched you pretty closely lately, Rachel, and taken a bit of trouble to find out what sort of a girl you really were, for you don't show it to everyone. And I've come to the conclusion that you will make a wife any man might be lucky and proud to have."

"I am glad you think that," she replied, her heart beating fast at this unusual praise, her eyes, with a happy light in them, looking straight into his.

"So you love me a bit too, Rachel, my dear," he continued, edging a little nearer to where she sat, and taking one of her hands in his, rather shyly and clumsily, for it was his first attempt at serious love-making, and Rachel was not the sort of girl to give a man a lead, or meet him half-way in his wooing.

"Yes, David, I do love you," she replied simply; many words or much protestation were not in her way.

They both—that pair of lovers on the green hill-top—forgot Esther waiting for them in the little cottage below, with the butter growing soft and oily in the heat, and the tea drawing in the little brown pot on the hob till it was fast becoming undrinkably black and bitter; while she fumed and fretted, and only because it would be such a long, hot climb, refrained from going up the hill herself, and putting an end to this objectionably long talk between David and Rachel.

"You won't go away now, Rachel?" observed David slyly, succeeding in getting his arm round her waist as he spoke.

In after-years that scene often rose up before the girl, as fresh and vivid in all its details as though it had happened yesterday.

She could see it all—the mist-shrouded valley, the crescent moon set high in the pale heavens, with one faint star shining near. She could see her lover's face, and hear his voice, and feel all that wonderful new joy at knowing that he cared for her. And if, by giving up those few minutes of intense happiness, she could have saved herself all the sorrows of her after-life, she would not have done it. No price was too high to pay for such rapture, brief and transitory though it might be.

"Well, I'm glad it is all settled, Rachel," said David, though he did not feel quite happy in his mind. There was an unpleasant quarter of an hour before him yet, when old Elias should be told the truth. David might then expect many cutting and sarcastic observations on his obstinacy; and though he did not choose to believe in his father's threat of ultimately disinheriting him, he knew the old man both could and would make things disagreeable for his son and daughter-in-law for some time to come.

"The sooner we settle down the better, my dear," he continued. "How soon could you be ready?"

"I could get everything straight in a week or two, David," she replied, a peculiar little smile playing about her mouth. "It won't take me long to get the clothes for *my* wedding; but I must make arrangements for Esther."

"She could live with us for a time," he answered magnanimously. "As you said before, it's not unlikely some young fellow may take a fancy to her and marry her before long. A pretty baby face like hers goes a great way with some people."

"Ah, David, I fear she won't like me to marry you; she cares too much for you herself."

"Well, it can't be helped," he said quickly, though he was gratified at the idea. It was pleasant to his vanity to think that two girls, at least, were desperately in love with him; and if two, why, there might be half-a-dozen more he did not know about. Really, Rachel ought to feel proud of having secured so coveted a prize. He was very sorry for Esther, too, poor little thing! He had cared very much about her once; and after all he might have magnified her apparent neglect of his attentions. He had Rachel's word for it that it was he Esther really loved, in spite of her other flirtations.

"Well, it can't be helped," he repeated. "It is you I am going to marry. When did you say it should be? In three weeks after the banns have been called?"

"No, David; never."

"What do you mean?" he said, rising to his feet and standing before her. "Do you mean to make a fool of me?"

"Yes," she replied, getting up in her turn, and confronting him with a white face and firm-set lips. "Yes, if you believe I mean to marry you."

"And after all you have said, you don't love me? You have only been laughing at me for being so easily taken in?"

"That is all," she replied, with a forced, harsh laugh that had no sound of mirth in it.

"Then I'll show you I don't care either!" he returned angrily. He faced round without another word, and began rapidly descending the hill towards the little cottage where Esther waited for him.

And Rachel, with broken lines, drooping, tattered banners, and gaping wounds, knew that after all she had gained the day, and won, at her own cost, the victory for her sister.

*(To be continued.)*

